# Trochantérite tuberculeuse isolée

**DOI:** 10.11604/pamj.2017.26.85.11631

**Published:** 2017-02-21

**Authors:** Zeineb Alaya, Walid Osman

**Affiliations:** 1 Service de Rhumatologie, Hôpital Farhat Hached, Sousse, Tunisie; 2 Service d’Orthopédie, Hôpital Sahloul, Sousse, Tunisie

**Keywords:** Trochantérite, tuberculose, scanner, scintigraphie osseuse, biopsie osseuse, Trochanteritis, tuberculosis, bone scintigraphy, bone biopsy

## Image en médecine

Un patient âgé de 40 ans, était hospitalisé pour exploration d'une douleur inflammatoire intéressant la face supéro-externe de la cuisse droite évoluant depuis 8 mois, sans altération de l'état général ni fièvre. La pression en regard du grand trochanter droit était douloureuse sans signes inflammatoires locaux. La biologie n'a pas montré de syndrome inflammatoire. La radiographie du bassin a révélé une lésion ostéolytique du grand trochanter droit avec épaississement des parties molles en regard (A). La scintigraphie osseuse a montré une hyperfixation du grand trochanter droit (B). Le scanner du basin a révélé une lésion ostéolytique du grand trochanter droit contenant de multiples séquestres, avec irrégularité corticale et collection multicloisonnée en regard de la lésion (C). Une ponction-biopsie osseuse du grand trochanter, sous contrôle scannographique (D), a mis en évidence un granulome épithélioïde et giganto-cellulaire à l'histologie, avec présence de BK à la culture du liquide de ponction. Le diagnostic de tuberculose trochantérienne était alors porté. L'intradermoréaction à la tuberculine (IDR) était négative. La recherche d'autres localisations tuberculeuses s'est révélée négative. Le patient a été mis sous antituberculeux pendant 12 mois avec bonne évolution. La tuberculose constitue actuellement une cause rare sinon exceptionnelle de douleur trochantérienne. La particularité de notre observation réside aussi dans l'absence d'autres localisations tuberculeuses.

**Figure 1:**
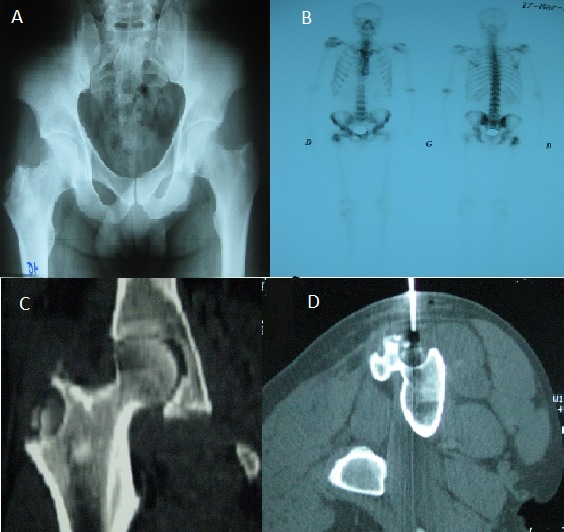
A): radiographie du bassin ; lésion ostéolytique du grand trochanter droit avec épaississement des parties molles en regard; B): scintigraphie osseuse; hyperfixation du grand trochanter droit; C): scanner du bassin: lésion ostéolytique du grand trochanter droit contenant de multiples séquestres, avec irrégularité corticale et collection multicloisonnée en regard de la lésion.; D): ponction-biopsie osseuse du grand trochanter sous contrôle scannographique

